# Photoactive imaging and therapy for colorectal cancer using a CEA-Affimer conjugated Foslip nanoparticle†

**DOI:** 10.1039/d3nr04118b

**Published:** 2023-11-03

**Authors:** Yazan S. Khaled, M. Ibrahim Khot, Radhika Aiyappa-Maudsley, Thomas Maisey, Arindam Pramanik, Jim Tiernan, Nicole Lintern, Eiman Al-Enezi, Shazana H. Shamsuddin, Darren Tomlinson, Louise Coletta, Paul A. Millner, Thomas A. Hughes, David G. Jayne

**Affiliations:** a Leeds Institute of Medical Research, St James's University Hospital Leeds United Kingdom Y.Khaled@leeds.ac.uk +44 113 2065281 +44 113 2065281; b School of Biomedical Sciences, University of Leeds Leeds UK; c Department of Pathology, School of Medical Sciences, University Sains Malaysia Malaysia; d School of Molecular and Cellular Biology, University of Leeds Leeds UK; e School of Medicine, University of Leeds Leeds UK; f School of Science, Technology and Health, York St John University York UK

## Abstract

Theranostic nanoparticles hold promise for simultaneous imaging and therapy in colorectal cancer. Carcinoembryonic antigen can be used as a target for these nanoparticles because it is overexpressed in most colorectal cancers. Affimer reagents are synthetic proteins capable of binding specific targets, with additional advantages over antibodies for targeting. We fabricated silica nanoparticles using a water-in-oil microemulsion technique, loaded them with the photosensitiser Foslip, and functionalised the surface with anti-CEA Affimers to facilitate fluorescence imaging and photodynamic therapy of colorectal cancer. CEA-specific fluorescence imaging and phototoxicity were quantified in colorectal cancer cell lines and a LS174T murine xenograft colorectal cancer model. Anti-CEA targeted nanoparticles exhibited CEA-specific fluorescence in the LoVo, LS174T and HCT116 cell lines when compared to control particles (*p* < 0.0001). No toxicity was observed in LS174T cancer mouse xenografts or other organs. Following photo-irradiation, the anti-CEA targeted particles caused significant cell death in LoVo (60%), LS174T (90%) and HCT116 (70%) compared to controls (*p* < 0.0001). Photodynamic therapy (PDT) at 24 h *in vivo* showed a 4-fold reduction in tumour volume compared to control mouse xenografts (*p* < 0.0001). This study demonstrates the efficacy of targeted fluorescence imaging and PDT using Foslip nanoparticles conjugated to anti-CEA Affimer nanoparticles in *in vitro* and *in vivo* colorectal cancer models.

## Abstract 2

Theranostic nanoparticles hold promise for simultaneous imaging and therapy in colorectal cancer.

## Graphical abstract

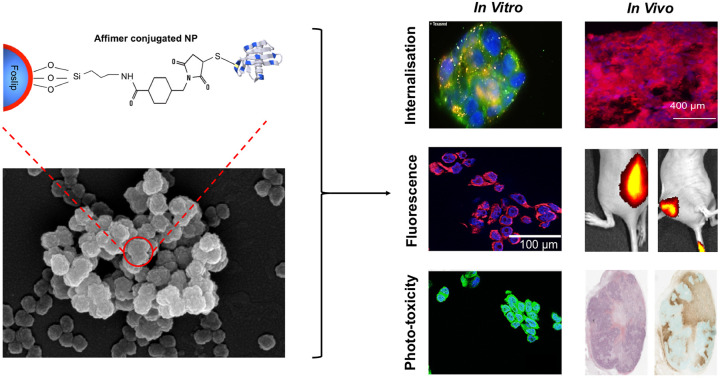

## Introduction

Personalised surgery involves a tailored approach for individual patients and underlying diseases. Up to 30% of colorectal cancer (CRC) patients undergoing curative surgical resection develop locoregional recurrence or distant metastases.^1–3^ Lymph node micrometastases and residual tumour cells are thought to be the main contributing factors. They are not detectable at surgery and can be easily missed during routine histopathological examination.^4,5^ An accurate means of identifying positive lymph nodes (LNs) intraoperatively would allow the radicality of surgery to be tailored to the biology of the primary cancer; lymph node positive cancers would undergo radical D3 lymphadenectomy, whereas lymph node negative cancers could be effectively treated by limited segmental resection. Reducing the radicality of surgery, whilst maintaining the oncological efficacy, is important as the incidence of CRC is rising, particularly amongst the elderly population.^6,7^

Theranostics has emerged as a promising route for personalised cancer treatment, allowing real-time imaging of cancers and cytotoxic cell killing.^8,9^ A theranostic photo-active nanoparticle would enable surgeons to visualise positive lymph nodes, tumour margins and distant metastasis intra-operatively, facilitating complete cancer eradication. Selective uptake of a photosensitiser (PS) by cancer cells allows fluorescence visualisation, whilst irradiation with a specific wavelength of light triggers cancer cell death due to generation of cytotoxic reactive oxygen species (ROS).^10–12^ Photodynamic therapy is particularly attractive in cancer surgery, which is now mostly undertaken using laparoscopy. Changing the light wavelength delivered to the abdominal cavity to activate a PS is relatively straightforward.

One of the few photosensitisers that is clinically approved for treatment of different cancers in Europe is *meta*-tetra(hydroxyphenyl)chlorin (mTHPC), commercially known as Foscan®.^13^ mTHPC is characterised by its favourable absorption wavelength in the near infra-red region (652 nm) and high singlet oxygen quantum yield.^14^ In preclinical studies, the liposomal formulation of mTHPC, known as Foslip®, gave enhanced PDT efficacy and offered several advantages such as being non-immunogenic and biodegradable in addition to increasing the drug solubility and tumour selectivity while reducing unwanted skin accumulation.^15,16^ However, a lack of stability, with 60% of liposome destruction after 24 hours, is an obvious shortcoming.^17–19^ Leakage of the phospholipid membrane can be halted by coating the liposomes with a polymer net^20^ or a silica shell^21^ and this is widely used to stabilise the lipid bilayer.^22^ Silica based nanoparticles are attractive because of their compatibility with biological systems and transparency to light. Their degradation is enhanced by the increased ROS levels observed in the tumour microenvironment, facilitating delivery of the payload. Silica-based ‘C dots’ have recently been approved for Phase 2 clinical trials.^23,24^ We have shown previously that carcinoembryonic antigen is a reliable tissue biomarker for colorectal cancer^25^ and that our CEA antibody targeted NIR664 dye-doped silica nanoparticles allowed specific *in vivo* fluorescence imaging of colorectal cancer in a mouse model.^26^ However, antibody-based drug targeting has its limitations, including high cost of production, stability, and batch-to-batch variation, which limit clinical translation.^27,28^ Affimers are an attractive alternative with equivalent biorecognition characteristics to antibodies.^29^ The absence of cysteine residues in the Affimer scaffold allows the introduction of cysteine for site-specific conjugation to nanoparticles. Affimers are thermo- and pH-stable and easily expressed in prokaryotic cells (*E. coli*), thereby reducing the cost of production. We have recently shown that CEA-Affimers bind to cancer cells expressing CEA with high affinity and with *K*_D_ values in the nM range.^30,31^

The aim of this study was to develop a photoactive, theranostic nanoparticle for fluorescence tumour imaging and ablation. We report the first successful use of Affimer-targeted, silica-coated Foslip nanoparticles for fluorescence imaging and photodynamic therapy *in vitro* and in an animal CRC model.

## Results

### Synthesis and characterisation of Affimer-tagged silica-coated Foslip nanoparticles

**Fig. 1:**
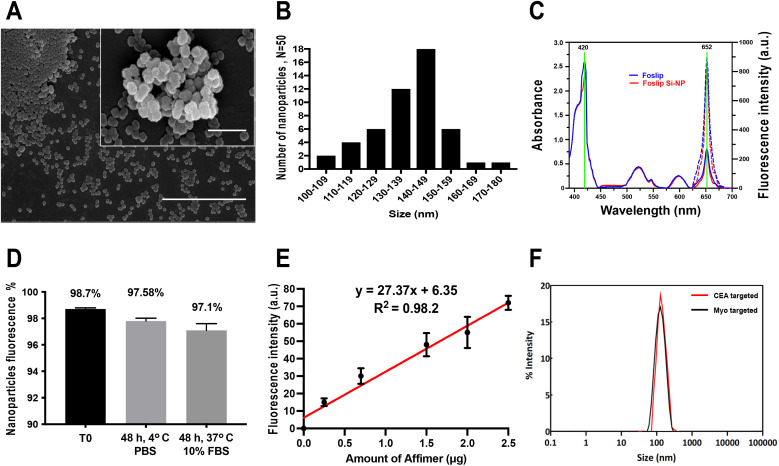
Characterisation of silica-coated Foslip NPs. (A) SEM image shows a spherical structure of NPs with a size of around 140 nm. The scale bars represent 1 μm and 500 nm respectively for the whole view and the magnified view. (B) The size distribution is shown with particle diameters being binned into 10 nm intervals. (C) UV absorption spectra of Foslip alone (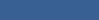) and silica-coated Foslip NPs (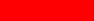); fluorescence spectra of Foslip alone (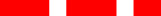) and silica-coated Foslip NPs (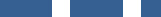). (D), Stability of Affimer-tagged NPs monitored at the fluorescence intensity under different conditions using a spectrofluorometer: the freshly prepared particles (T0); the sample stored for 48 h at 4 °C in PBS; the sample incubated at 37 °C in 10% FBS for 48 h. Data denote mean from 3 biological experiments (SEM, *n* = 3). (E) A calibration curve of the fluorescence intensity for the NanoOrange dye with the increasing dose of Affimer concentration. Data denote mean from 3 biological experiments (SEM, *n* = 3). (F) Affimer-tagged NP size as determined by DLS.

We aimed to synthesise silica-coated Foslip nanoparticles to target colorectal cancer cells using anti-CEA Affimers as bioreceptors. Silica coating formation was achieved by a hydrolysis process of TEOS, according to protocols published in the literature.^32,33^ The precipitation of silica on the surface of Foslip resulted in the formation of a spherical core–shell-like structure, as visualised by scanning electron microscopy (Fig. 1A), with a mean diameter of 140 nm (±1 nm SEM) (Fig. 1B). Encapsulation of Foslip was demonstrated by absorption and fluorescence emission spectra of the synthesised nanoparticles. Fluorescence of the nanoparticles was measured and recorded using a Cary Eclipse spectrofluorometer in water suspension, using specific Foslip excitation and emission wavelengths of 420 and 652 nm, respectively.^34^Fig. 1C shows a typical spectrum of silica-coated Foslip particles, along with a spectrum of Foslip alone, demonstrating that encapsulation does not alter the spectral properties. The efficiency of the encapsulation (EE) process was quantified by measuring the absorbance of Foslip with reference to a dose standard curve using a microplate reader (Fig. S1†). A typical nanoparticle sample contained 1 mg ml^−1^ nanoparticles and 110 nM Foslip correlated with Foslip ee of ∼82.2 ± 2.1% (*n* = 3). We also assessed the stability of nanoparticles under different conditions by measuring their fluorescence using a plate reader, as shown in Fig. 1D. Particles remained highly fluorescent either in stock PBS at 4 °C (98.5%) or in PBS containing 10% (v/v) FBS at 37 °C (97.0%) for 48 h, when compared to freshly synthesised NPs, after which the signal reduced most likely due to the leakage of mTHPC; Fig. S2.† Despite this limitation, the NPs were able to achieve their target imaging and cytotoxicity in less than 48 h as shown in the following results.

Next, we selected two different anti-CEA Affimers (based on protein yield) to achieve polyclonal targeting of the CRC-antigen CEA, and as a control, an anti-myoglobin (Myo) Affimer. The anti-myoglobin Affimer was used as a negative control because colorectal cancer cells do not express this human cardiac muscle-related protein. Tris (2-carboxyethyl) phosphine (TCEP) reduced anti-CEA, and anti-Myo Affimers were purified using Ni^2+^-NTA resin (Fig. S3 and Table ST1†) and prepared for conjugation. The hetero bifunctional cross-linker sSMCC (sulfosuccinimidyl 4-(*N*-maleimidomethyl) cyclohexane-1-carboxylate) was then used to link the free Affimer sulfhydryl group to the aminated nanoparticle surface (provided by the salinisation agent, 3-aminopropyl triethoxysilane (APTES)) as shown in Fig. S4.†

Quantification of the Affimer amount bound to NPs comprised two main parts: (i) cleavage of the disulfide bond cross-linked between the Affimer and NPs and (ii) quantification of the Affimer concentration. The Affimer-tagged NPs were prepared at 1 mg mL^−1^ concentration and the reducing agent 2-mercaptoethanol was used to break the thiol-maleimide conjugation and free the Affimers. Free Affimers were recovered from the supernatant and the concentration was measured using a calibration curve for NanoOrange® protein; Fig. 1E. Knowing the estimate number of NPs per mL (∼8.5 × 10^7^), the number of Affimers immobilised on each NP was estimated at 570 ± 110 Affimer/NP.

Dynamic light scattering (DLS) showed monodisperse particle peaks at 148 nm (±11 nm) and reassuringly, CEA-Fos-NPs and Myo-Fos-NPs showed almost identical size distributions; Fig. 1F. The mean zeta potential of the silica-coated Foslip exhibited a negative surface charge (−15.6 mV), whereas that of aminated NPs exhibited a positive surface charge (27.9 mV). The surface charge of the Affimer-tagged NPs shifted back to a more neutral charge state (2.8 mV) indicating successful conjugation. The size distribution, zeta potential and polydispersity index (PDI) of NP derivatives are shown in Table 1.

**Table 1:** Size distribution, zeta potential and PDI data of NP derivatives

Batch	*Z*-average hydrodynamic diameter (nm)	Zeta potential (mV)	PDI
Si-Fos-NP	138 (±4 nm)	−15.6 (± 4.2 mV)	0.21
Aminated Si-Fos-NP	140 (±2 nm)	27.9 (± 12.4 mV)	0.24
CEA/Myo-Fos-NP	148 (±1 nm)	2.8 (± 1.1 mV)	0.19

### CEA-Fos-NPs enabled selective fluorescence imaging and cytotoxicity in colorectal cancer cell lines

**Fig. 2:**
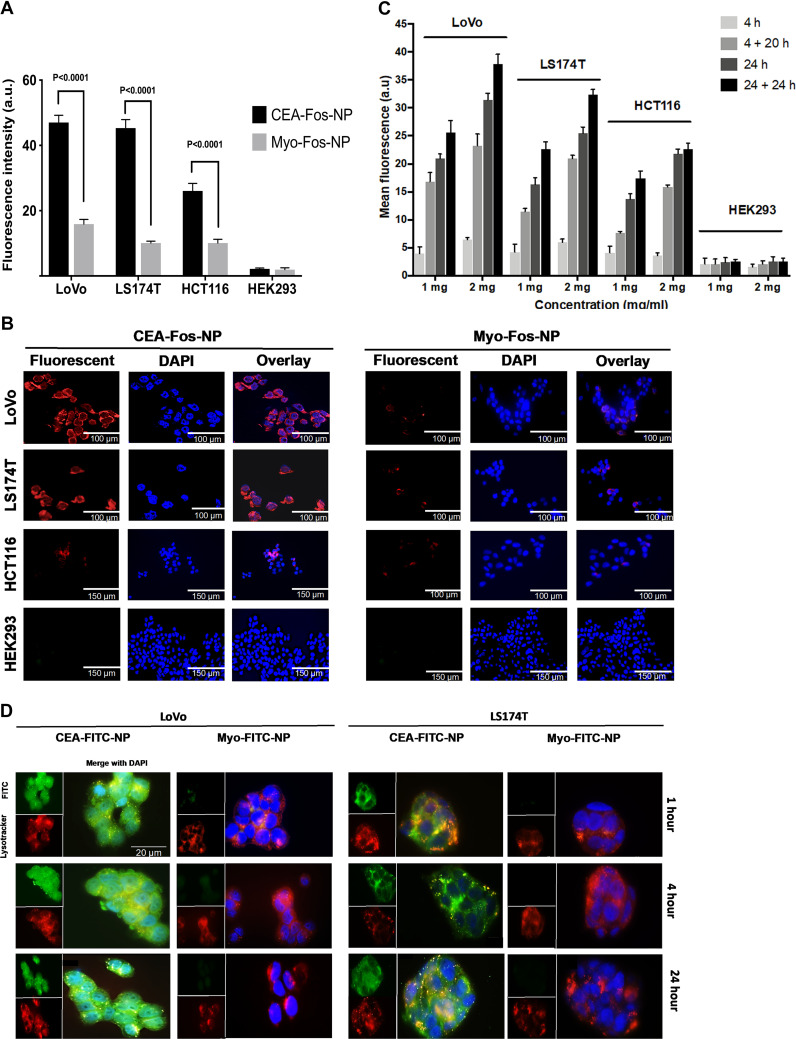
CEA-Fos-NP enabled targeted fluorescence imaging of CRC cells. (A) Comparing the fluorescence intensity in CRC cancer cells when incubated with nanoparticles conjugated to either CEA-Fos-NPs or Myo-Fos-NPs for 24 h. Data denote mean fluorescence from 3 biological experiments (SEM, *n* = 3). Significance was tested using unpaired *t*-tests. (B) Representative confocal images of LoVo, LS174T, HCT116 and HEK293 cells right after 24 h incubation with CEA-Fos-NPs or Myo-Fos-NPs. (C) Cellular uptake of CEA-Fos-NPs in CRC and control cell lines at different time points and nanoparticle concentrations. Cells were incubated with 1 or 2 mg mL^−1^ of anti-CEA targeted nanoparticles for 4 and 24 h. After incubation, the cells were washed and fresh nanoparticle-free media were added for additional 20 h (4 + 20 h) and 24 h (24 + 24 h). Data denote mean fluorescence from 3 biological experiments (SEM, *n* = 3). (D) Fluorescence images of CEA-FITC-NPs internalised into the cytoplasm and lysosomes of LoVo and LS174T. Images show FITC from NPs (green, top left), LystoTracker staining of lysosomes (Red, bottom left) and merged DAPI fluorescence of NPs and lysosomes (yellow, magnified). Scale bar is 20 μm for all images.

We aimed to assess the fluorescence and PDT effect on three colorectal cancer cell lines (LoVo, LS174T and HCT116) and a control, CEA-negative non-cancer cell line (HEK293), when incubated with CEA-Fos-NPs. We have previously reported that LoVo cells show high CEA expression, LS174T cells show moderate to high CEA expression, HCT116 cells show low CEA expression, and HEK293 cells show no expression of CEA.^26^ Anti-CEA or Myo-Affimer tagged nanoparticles (1 mg mL^−1^) were incubated with colorectal cancer and control cell lines for 24 h and then imaged using confocal microscopy, and cell-specific fluorescence was quantified. CEA-Fos-NPs exhibited more intense tumour-specific targeting, with anti-CEA targeted nanoparticles showing 9.5-, 10.2- and 3.5-fold greater fluorescence than Myo-Affimer targeted nanoparticles in LoVo, LS174T and HCT116 cells respectively (*p* < 0.0001) as shown in Fig. 2A. Importantly, CEA-Fos-NPs did not produce any significant fluorescence intensity in the control cell line HEK293, suggesting that the anti-CEA Affimer targeted silica nanoparticles were specific to CEA expressing cells and likely to prevent unwanted accumulation in normal tissues, thereby reducing side effects. Representative confocal microscopy images showed fluorescence in tumour cells at 24 h that correlates with CEA expression data in the literature (Fig. 2B). In order to assess the dose- and time-effect on cellular fluorescence, cells were incubated with 1 or 2 mg mL^−1^ nanoparticles for 4 and 24 h. After incubation, the cells were washed and fresh nanoparticle-free media were added for an additional 20 h (4 + 20 h) or 24 h (24 + 24 h). Spectrofluorometer evaluation showed that LoVo cells had significantly higher fluorescent signal with CEA-Fos-NPs than other cell lines (*p* < 0.02), followed by LS174T and HCT116 cells, in a dose and time dependent manner (Fig. 2C). Cellular uptake was seen as early as 4 h but the difference between the single time points was most obvious at 24 and 24 + 24 h in all cancer cell lines. Although fluorescence was still present in cells after 24 h incubation, the mean fluorescence intensity at 24 + 24 h was greater than 24 h (*p* = 0.01), indicating that cellular uptake was also time dependent. The mean fluorescence in HEK293 cell lines was almost identical at 4 + 20 h and 24 h (*p* > 0.9), whilst in the colorectal cell lines a significant difference was observed between these two time points (*p* < 0.001), highlighting that the anti-CEA Affimer increased the selectively for cancer cells.

Next, we assessed the internalisation and co-localisation characteristics of NPs in LoVo and LS174T cells. For the purpose of this experiment, we synthesised silica NPs tagged with fluorescein isothiocyanate (FITC) and CEA or Myo-Affimer. NPs (1 mg mL^−1^, 150 nm (±12 nm)) were incubated with cells for 1, 4 and 24 hours and then imaged by fluorescence microscopy to track internalisation. To determine the subcellular localisation of NPs, we subsequently stained cells with LysoTracker Deep Red. Based on fluorescence microscopy images, CEA-FITC-NPs were internalised in LoVo and LS174T as early as 1 h whereas Myo-FITC-NPs were negligibly internalised. The CEA-targeted NPs predominantly accumulated into the cytoplasm, with some lysosomal localisation as shown in Fig. 2D, where the LysoTracker (red) and the nanoparticles (green) were co-localised (yellow).

Next, we assessed the dark cytotoxicity of CEA-Fos-NPs against colorectal cancer cells. Cells were incubated with CEA- or Myo-Fos-NPs at a high concentration of 3 mg mL^−1^ for 24 h and 24 + 24 h, then washed and kept in nanoparticle-free media followed by MTT assay quantification of cellular viability. Cells were kept in the dark during incubation periods. Affimer tagged Fos-NPs did not affect the survival of all cell lines when exposed at a high concentration of 3 mg mL^−1^ for 24 h, which is much higher than that used to achieve cell-specific fluorescence and cellular uptake in previous experiments. Similarly, the MTT assay showed that the number of metabolically active cells at 24 + 24 h after exposure to nanoparticles was not reduced relative to controls (Fig. S5†).

We assessed the light-dose effect on cell survival to ensure that any cytotoxic effect was Foslip-mediated only. Cells were incubated with CEA-Fos-NPs at various concentrations for 24 h, washed and incubated with fresh media, and immediately incubated in the dark (0 J cm^−2^) or photo-irradiated with light doses from 0.15 to 0.675 J cm^−2^. Cells were then kept in nanoparticle-free media for an additional 24 h followed by assessment of cell viability by MTT assay.

**Fig. 3:**
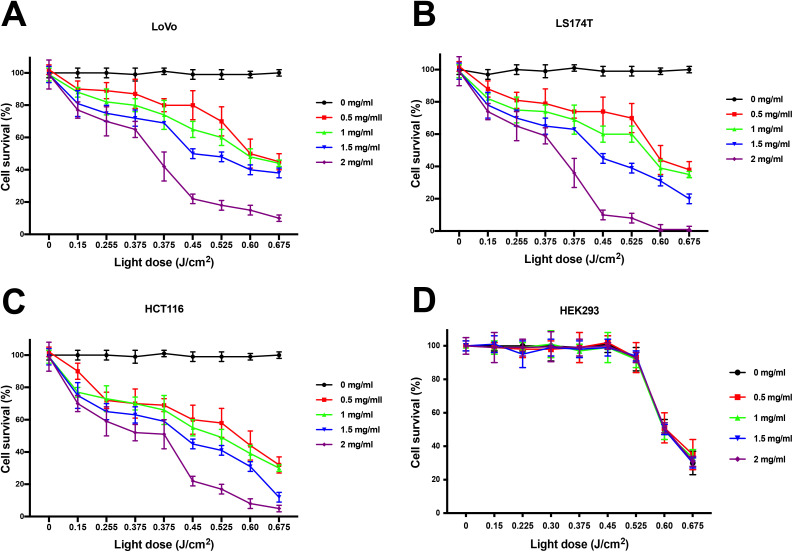
Light dose and nanoparticle concentration effect on CRC cells. (A–D) Cells were incubated with various concentrations of CEA-Fos-NPs for 24 h and then photo-irradiated with 0.15–0.675 J cm^−2^ at 600–700 nm. Cells viability was quantified using MTT assay. Data denote mean cell viability from 3 biological experiments (SEM, *n* = 3).

In all the cancer cells, significantly reduced viability of cells was observed that was dependent on light dose and on nanoparticle dose (*p* < 0.0001; Fig. 3A–C), with no reduction in viability in the absence of nanoparticles at any light dose. For example, more than 80% reduced viability was seen at the highest doses of nanoparticles after 0.45 J cm^−2^ irradiation. By contrast, HEK293 cells showed no reduction in viability at any dose of nanoparticles below 0.6 J cm^−2^ (Fig. 3D); at 0.6 J cm^−2^ and above, HEK293 cells showed light-induced toxicity that was independent of the presence of nanoparticles suggesting that these cells were more sensitive to light alone than the cancer cells. Therefore, light dose at 0.45 J cm^−2^ was considered as the cut-off point for safe photo-irradiation of cells in the next experiment.

**Fig. 4:**
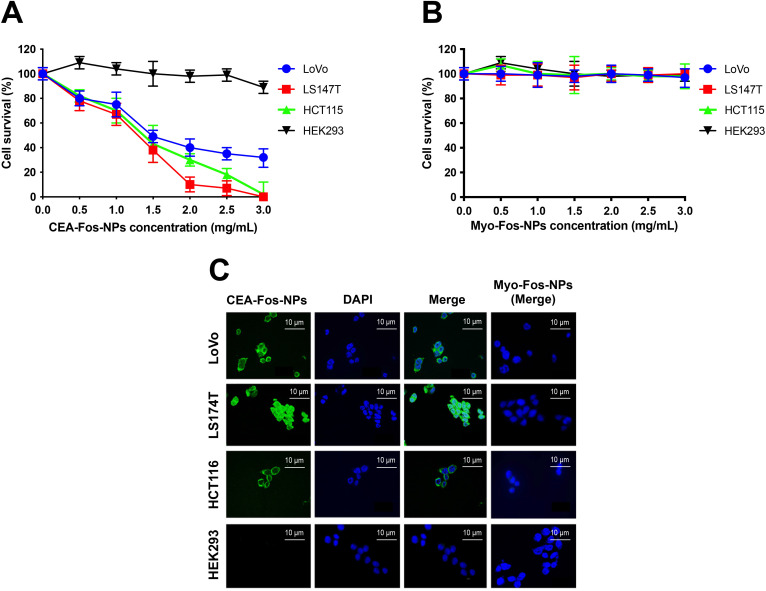
CEA-Fos-NP enabled targeted PDT in CRC cells. (A–B), Cells were incubated with various concentrations of CEA-Fos-NPs or Myo-Fos-NPs respectively for 24 h and then photo-irradiated with 0.45 J cm^−2^ at 600–700 nm. Cells viability was quantified using MTT assay. Data denote mean cell viability from 3 biological experiments (SEM, *n* = 3). (C) Representative confocal images of ROS detection in cells following PDT post-incubation with nanoparticles at 2 mg mL^−1^ concentration and 0.45 J cm^−2^ light dose are shown. Cells were incubated with PBS containing 10 mM DCFDA for 30 minutes in a CO_2_ incubator and then washed with PBS. DCF fluorescence observed in cells and their respective DAPI staining are shown. Confocal images of ROS detection showed DCF fluorescence in cancer cells treated with CEA-Fos-NPs but not with Myo-Fos-NPs.

Next, we assessed the phototoxicity efficacy of CEA-Fos-NPs in killing cancer cells when photo-irradiated with the optimum light dose of 0.45 J cm^−2^. Cells were incubated with CEA-Fos-NPs or Myo-Fos-NPs at various concentrations for 24 h, washed and incubated with fresh media, and immediately photo-irradiated with light doses of 0.45 J cm^−2^ followed by MTT assay assessment as described previously. As shown in Fig. 4A, at 0.45 J cm^−2^ light dose, a significant reduction in cell survival was observed in LoVo, LS174T and HCT116 cells when compared to control HEK293 cells (*p* < 0.0001). The reduction in cell survival measured at 24 h after irradiation with the optimum light dose of 0.45 J cm^−2^ was dose dependent. At 2 mg mL^−1^ CEA-Fos-NP concentration, significant cell death was observed in LoVo (60%), LS174T (90%) and HCT116 (70%) when compared to that in HEK293 (0%); *p* < 0.0001. Importantly, no cellular toxicity was observed when cells were treated with increasing dose of the control anti-myoglobin Affimer nanoparticles at 0.45 J cm^−2^ (Fig. 4B). Interestingly, the PDT induced cellular toxicity did not correlate with the fluorescence intensity seen in the respective cell lines as shown earlier in Fig. 2. As the cell density per well may impact the overall PDT efficacy, we attempted to standardise this variable by measuring cell viability per 1000 cells per well. Following PDT, cells were trypsinised and stained with trypan blue, and the number of viable cells per 1000 cells per well was calculated (Fig. S6†). The data show that when cell numbers were standardised per well, LoVo cell viability dropped to ∼30%. Importantly, the variation in response to PDT appeared to correspond to the degree of differentiation of the cell line suggesting that tumour cell biology and genetic differences may be associated with variations in cellular pathways and overall sensitivity to PDT.

The DCFDA assay was performed to study the cell death mechanism following PDT to mimic the experiments in which cell viability was assessed using the MTT assay. DCF fluorescence was observed using a confocal microscope. The assay showed strong fluorescence in all cancer cells treated with CEA-Fos-NPs but not with Myo-Fos-NPs or in control cells (Fig. 4C). The results support the hypothesis that the cytotoxic effect seen in the PDT experiment was Foslip mediated *via* ROS generation. Collectively, Foslip-loaded silica nanoparticles conjugated to anti-CEA Affimers allowed tumour cell-specific fluorescence and photodynamic therapy *in vitro*.

### Theranostic application of CEA-Fos-NPs in the LS174T xenograft model of colorectal cancer

**Fig. 5:**
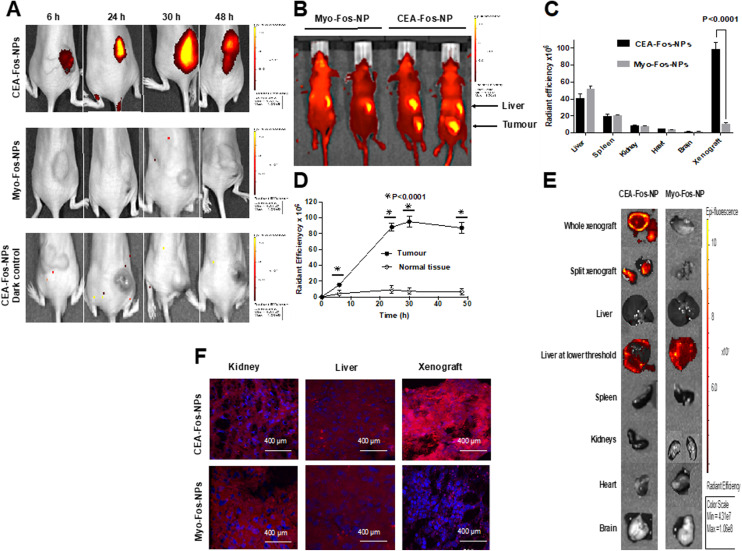
CEA-Fos-NP enabled targeted fluorescence imaging of CRC *in vivo.* (A) Representative *in vivo* fluorescence activation of CEA-targeted *versus* control NPs in the LS174T xenograft model at 6, 24, 30 and 48 h after intravenous injection with 150 μL (2 mg mL^−1^), *n* = 5. Colour scale bar: minimum = 4.31 × 10^7^ and maximum = 1.06 × 10^8^ (p s^−1^ cm^−2^ sr^−1^)/(μW cm^−2^). (B) Fluorescence *in vivo* biodistribution of CEA-targeted and control NPs at 24 h post NP injection. (C) NP biodistribution quantified using IVIS with excitation filters at 615–665 nm and 8 s exposure time. Data denote mean fluorescence (SEM, *n* = 5). (D) Data are mean tumour fluorescence of *in vivo* xenografts for mice injected with CEA-Fos-NPs and Myo-Fos-NPs (SEM, *n* = 5). Normal tissue represents skin. (E) Representative *ex vivo* fluorescence images of the excised organs and xenografts from CEA-targeted and control dosed mice at 24 h post injection. The liver at lower threshold = (∼50 × 10^6^ (p s^−1^ cm^−2^ sr^−1^)/(μW cm^−2^)). Colour scale bar: minimum = 4.31 × 10^7^ and maximum = 1.06 × 10^8^ (p s^−1^ cm^−2^ sr^−1^)/(μW cm^−2^). (F) Fluorescence histology images of the kidney, liver and tumour xenografts from mice injected with CEA-Fos-NPs and Myo-Fos-NPs 48 h after injection using confocal microscopy.

We next assessed the theranostic potential of CEA-Fos-NPs in a clinically relevant mouse xenograft model of colorectal cancer. The tumour growth pattern of LS174T xenograft is shown in Fig. S7.† Nanoparticles were suspended in sterile PBS at 2 mg mL^−1^ concentration and 150 μL of nanoparticles was injected into the tail vein of mice. Two groups of five mice were injected with either CEA-Fos-NPs (*n* = 5) or control Myo-Fos-NPs (*n* = 5) and imaged at 6, 24, 30 and 48 h. For better understanding of the biodistribution and fate of the nanoparticles, one mouse from each group was sacrificed after imaging at each time point, and organs were harvested and then imaged *ex vivo*. The background fluorescence point was set high to eliminate hepato-biliary fluorescence and ensure that any fluorescence seen in the xenograft was a real signal. Tumour-specific fluorescence was seen in the xenografts of mice that were injected with CEA-Fos-NPs as shown in Fig. 5A. No fluorescent signal was seen in any of the mice that were injected with Myo-Fos-NPs. The fluorescent signal was seen as early as 6 h, peaked at 24–30 h and remained in the xenograft at 48 h. When background fluorescence was set to a lower point (∼50 × 10^6^ (p s^−1^ cm^−2^ sr^−1^)/(μW cm^−2^)), Foslip loaded nanoparticles exhibited a similar biodistribution to our previously published report on NIR664-dye-doped silica nanoparticles;^26^Fig. 5B and C. Liver fluorescence was evident at 6 h in all mice (mean 59.1 × 10^6^ (p s^−1^ cm^−2^ sr^−1^)/(μW cm^−2^)) and increased at 24 h (85.8 × 10^6^ (p s^−1^ cm^−2^ sr^−1^)/(μW cm^−2^)). Hepatic localisation was confirmed by *ex vivo* imaging of isolated organs. There was no significant difference in liver fluorescence between mice injected with control particles and those injected with anti-CEA Affimer targeted particles at any time point; Fig. S8.†

Fluorescence in the CEA-targeted tumours was significantly greater than that in Myo-targeted tumours at all time points (*p* < 0.0001). Mean tumour fluorescence increased from 6 h (mean 0.55 × 10^7^ (p s^−1^ cm^−2^ sr^−1^)/(μW cm^−2^)) to 30 h (mean 9.415 × 10^7^ (p s^−1^ cm^−2^ sr^−1^)/(μW cm^−2^)); Fig. 5D. The fluorescence ratio, which was defined as the fluorescence of the tumour site over the fluorescence of normal tissue, at 6, 24, 30 and 48 h was 21, 88, 95 and 85 respectively. No tumour fluorescence, above background, was seen in mice injected with Myo-Fos-NPs.

Tumour tissue, and other organs, were harvested and imaged *ex vivo*. Tumour fluorescence was only detected in xenografts from mice injected with CEA-Fos-NPs, as shown in Fig. 5E. Importantly, the *ex vivo* imaging of the CEA-targeted xenografts showed fluorescence within the core of the xenograft, suggesting that the nanoparticles accumulated within the tumour microenvironment. To confirm this, confocal imaging was performed on histological sections from a xenograft of a mouse injected with CEA-Fos-NPs and Myo-Fos-NPs; Fig. 5F. The xenograft showed the fluorescent signal within the tumour (section taken through the middle of the xenograft) whilst no fluorescence was seen in the control xenograft, suggesting directed-nanoparticle delivery to the tumour site.

**Fig. 6:**
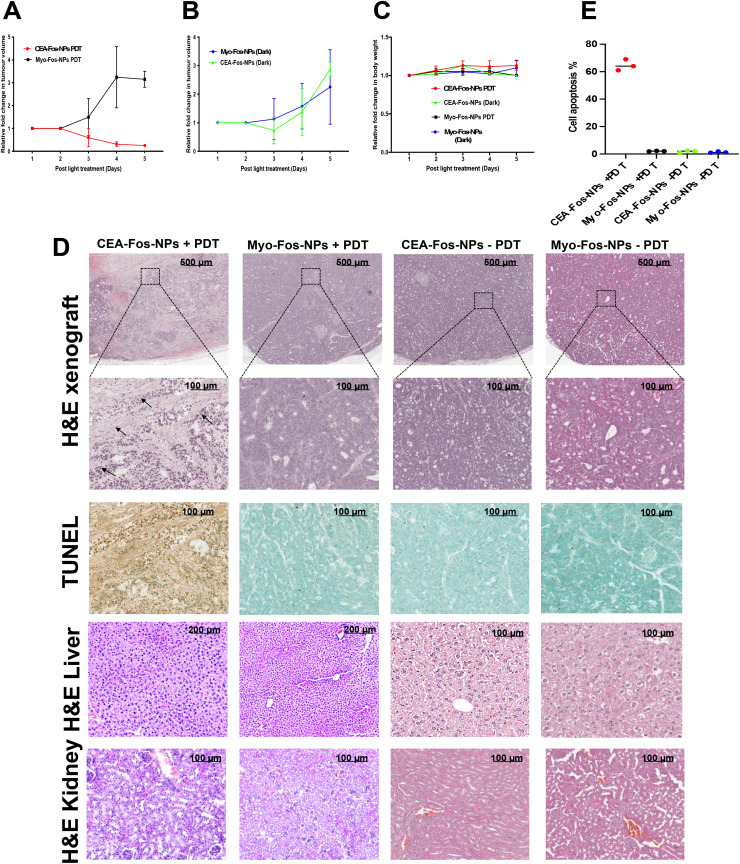
CEA-Fos-NP enabled targeted PDT of CRC *in vivo.* (A) Tumour growth curves of PDT groups over the treatment period until mouse sacrifice (SEM, *n* = 5). (B) Tumour growth curves of control dark groups over the treatment period until mouse sacrifice (SEM, *n* = 5). (C) Body weight curves of different groups over the treatment period until mouse sacrifice, (SEM, *n* = 5). (D) Representative images of histological analyses of tumour sections (H&E and TUNEL staining), the liver and the kidneys (H&E) at day 5 post treatment. Black arrows point to condensed nuclei and loss of cell structure. (E) Quantitative analysis of TUNEL positivity out of the whole tumour region in the four groups (*n* = 3).

Having observed significant accumulation of CEA-Fos-NPs in colorectal tumours, we next wished to test whether they could mediate efficient PDT activity *in vivo*. A further *in vivo* experiment was performed: xenograft tumours were established as before and animals were split randomly into four groups (each *n* = 5). Two groups were treated with CEA-Fos-NPs and with Myo-Fos-NPs as previously and then subjected to PDT. Two control groups were treated with CEA-Fos-NPs or Myo-Fos-NP and received no PDT. PDT, given 24 h post-delivery of NPs, consisted of transcutaneous laser irradiation (650 nm, 60 mW cm^−2^, 50 J cm^−2^, 14 min). The PDT efficacy was evaluated by tumour volume measurements and postmortem histopathological analysis. To eliminate the possibility of laser-induced thermal ablation and cell death in xenografts, thermal imaging videos were recorded for 1 min at 0 min, 7 min and 14 min during treatment for each mouse and we found no noticeable increase in surface temperature during laser treatment (Fig. S9†). The CEA-targeted PDT group displayed an ∼4-fold decrease in tumour volume when compared to the Myo-targeted PDT group at day 5 (0.24 *vs.* 3.15 median; *P* < 0.001) whilst mouse weights remained unchanged in all groups; Fig. 6A–C. Importantly, there was no reduction in tumour volume in any of the dark control groups. Histological analysis showed condensed nuclei and loss of cell structure in the tumour xenografts of CEA-targeted PDT, which were not observed in control groups; Fig. 6D. TUNEL assay revealed dense staining (brown) at the site of DNA fragmentation in CEA-Fos-NP PDT xenografts in keeping with significant tumour cell apoptosis (64 ± 2.3%), whilst the controls showed methyl green of normal cells, indicating no tumour apoptosis (2 ± 0.1% for Myo-Fos-NPs, 1.8 ± 0.5% and 1.3 ± 0.3% for PDT-negative controls) (Fig. 6E). Overall, the results demonstrate the high selectivity and accuracy of CEA-targeted PDT to colorectal tumour xenografts.

## Discussion

Developing a targeted nanoparticle against a specific tissue to produce a reliable molecular probe remains challenging. Several studies have demonstrated improved delivery when a nanoparticle is actively targeted to CEA in colorectal cancer cells using site specific reagents such as antibodies, antibody-fragments, aptamers, peptides and nanobodies.^35–39^ To date, the anti-CEA antibody has shown the most promising targeting bioreceptor in colorectal murine models but translation to clinical application was complicated by the immunogenicity and the clearance from bloodstream, both owing to binding of Fc receptor-containing entities to the antibody Fc region.^40^ In addition, antibody size (∼150 kDa) makes cell penetration difficult. Tiernan *et al.* (2015) were the first to show specific tumour fluorescence imaging using NIR669-doped silica nanoparticles (mean diameter of 65 nm) in the LS174T murine xenograft mouse model.^26^ They immobilised monoclonal anti-CEA antibody to the surface of the nanoparticle using the PAMAM dendrimer. Conjugation of anti-CEA Affimer carried out with sSMCC, as used here, showed strong tumour-specific targeting in the same animal model. However, anti-CEA Affimer targeted xenografts exhibited higher fluorescence mean 9.415 × 10^7^ (p s^−1^ cm^−2^ sr^−1^)/(μW cm^−2^) *vs.* 4.74 × 10^7^ (p s^−1^ cm^−2^ sr^−1^)/(μW cm^−2^) and a similar biodistribution but with notably lower liver uptake. In keeping with our findings, Pramanik *et al.* (2022) have shown that anti-CEA Affimer tagged cubosomes, loaded with copper acetylacetonate as a model drug, actively targeted LS174T colorectal cancer cells *in vivo.*^41^ The authors showed preferential accumulation in colorectal cancer mouse xenografts, while maintaining low nonspecific absorption and toxicity in other organs. Owing to their small size (∼12 kDa), controlled orientation on the surface of nanoparticles and their high affinity to CEA expressing cells (*K*_D_ = 15.3 ± 0.37 nM and 34.4 ± 16 nM for the two Affimers tested),^30^ anti-CEA Affimers are expected to achieve important advances in CEA-targeting nanotechnologies. Despite the huge potential of new tools, targeting the CEA biomarker, research into CEA-targeting systems to enhance the efficiency of colorectal cancer targeting has been limited.

High accumulation and penetration of anti-cancer drugs into the inner parts of the tumour tissues are required to efficiently eradicate malignancies. Our data showed that anti-CEA Affimer targeted nanoparticles allowed significant Foslip-mediated localisation in tumour cells and photodynamic therapy *in vitro* and *in vivo* when compared to control nanoparticles. Reports on targeted delivery of mTHPC nanoparticulate formation to colorectal cancer cells for photodynamic therapy are limited. Millard *et al.* (2020) used ∼203 nm mTHPC-loaded extracellular vesicles (EV) and compared them with Foslip in a colorectal HT29 murine xenograft mouse model.^42^ They showed that in 3D cancer cell models, mTHPC-EV uptake caused deeper penetration after 24 h incubation as compared to Foslip, whilst the *in vivo* results showed a 33% increase in tumour killing with PDT treatment applied 24 h after injection but 0% was observed after Foslip-mediated PDT. However, a concerning finding was the big difference between liposomal and EV formulations in mTHPC-EV accumulation in the lungs (five to seven times higher than Foslip) and the liver. In sharp contrast, our data showed that significant fluorescence was only observed in the hepatobiliary system, which peaked at 24 h and reduced by 48 h post-injection. Other studies that investigated the biodistribution and excretion of silica nanoparticles have reported similar findings.^43–45^ Bretin *et al.* (2019) demonstrated the usefulness of nanoparticle encapsulation for PDT tumour targeting efficacy in CRC.^46^ They used 80 nm silica nanoparticles coated with xylan to encapsulate 5-(4-hydroxyphenyl)-10,15,20-triphenylporphyrin (TPPOH) and tested it in a colorectal HT-29 murine xenograft mouse model. They showed significant phototoxic effects of TPPOH-X SNPs mediated by ROS generation and stronger cell uptake in human colorectal cancer compared to free TPPOH. Abdelghany *et al.* (2013) successfully encapsulated the *meso*-tetra(*N*-methyl-4-pyridyl) porphine tetra tosylate (TMP) photosensitiser in a hydrogel-based chitosan/alginate nanosystem with an anti-death-receptor-5 (DR5) antibody tagged onto the surface.^47^ Although their nanoparticle elicited a more potent phototoxic effect than a free drug, the nanoparticle diameter was prohibitively large at 560 nm. In addition, DR5 is not specific to colorectal cancer and is not highly expressed. Others have reported successful encapsulation of mTHPC in nanoparticles for photodynamic therapy in colorectal cancer cells, but without a surface targeting molecule.^48^ To date, Foslip has been intensively tested in different *in vitro* preclinical models (2D and 3D tumour cell cultures),^34,49,50^ whilst PDT studies, including biodistribution, pharmacokinetics, and PDT efficacy in tumour-bearing *in vivo* animal models, are limited.^51,52^

The surgical management of colorectal cancer is often limited by difficulty in delineating tumour margins and an inability to visualise occult nodal metastasis. This predisposes to tumour recurrence and decreased survival. Although several studies have investigated the efficacy of PSs for PDT regimens in CRC, only a few studies have meticulously explored their application for fluorescence imaging. We have shown that CEA-Fos-NPs enabled real-time fluorescence imaging of colorectal tumours, accurately distinguishing tumour from normal tissue. Gavrina *et al.* (2018) investigated Chlorin e6 (Ce6) conjugated to polyvinyl alcohol (PVA) nanoparticles for *in vivo* fluorescence imaging in the CT26 xenograft model.^53^ The authors found a higher tumour-to-normal signal in mice treated with Ce6–PVA nanoparticles when compared to Ce6 alone. Xu *et al.* (2018) fabricated H_2_S-responsive NIR-fluorescent silica based NPs which allowed fluorescence imaging in HCT116 CRC cells, both *in vitro* and *in vivo.*^54^ The study design lacked control NPs and control cell lines, while for the *in vivo* experiment, the NPs were injected into the core of the tumour xenograft and it was not systemic. Soster *et al.* (2012) used PEG-conjugated dye-doped silica nanoparticles *via* systemic delivery to image CRC metastases in murine xenograft models.^55^ They used ‘bare’ nanoparticles as controls and imaged fluorescence only in *ex vivo* organs, raising concerns for antigen-specific targeting.

## Conclusion

We have successfully developed a unique targeting strategy to deliver Foslip to colorectal cancer cells in an animal model using a novel Affimer protein. We have shown that Affimer-tagged silica-coated Foslip nanoparticles are effective theranostic agents. The nanoparticle design enables stable assembly of the components within a small-sized structure, with a favourable pharmacokinetic profile and biodistribution and superior cellular uptake. Our nanoparticle is potentially applicable to targeting other solid tumours by changing the surface Affimer, provided that there is a specific tissue biomarker.

## Materials and methods

### Synthesis of silica coated Foslip nanoparticles

All experiments were performed at room temperature using Sigma Aldrich (USA) reagents unless otherwise stated. Nanoparticle synthesis was modified from previous publications.^22,26^

The water soluble *meta*-tetra (hydroxyphenyl) chlorin (mTHPC) Foslip® photosensitiser (20 mg mL^−1^ DPPC/DPPG, 2.2 mM mTHPC, 50 mg mL^−1^ glucose) was provided by Biolitec AG (Jena, Germany) with a molecular weight of 680.764 g mol^−1^. The powder was dissolved in PBS to make a stock solution of 100 μM and filtered through a syringe filter (0.1 μm pore size; TPP, Trasadingen, Switzerland). Tetraethyl orthosilicate (TEOS) was added (12 μL) to 1 mL of deionised water and stirred at 200 rpm for 24 h at room temperature. Next, 20 μL of the Foslip suspension was added to the TEOS solution and the mixture was stirred at 200 rpm for 1 h. Two mL of PBS (1×) buffer solution was added to the mixture and stirred for 30 min, then 24 μL of fresh TEOS were added and the mixture was stirred at 200 rpm for 48 h. The mixture was then transferred into Corex centrifuge tubes (Corning) with equal volumes. Particles were pelleted by centrifugation (15 000*g*, 25 min), resuspended in wash solution using ultrasound sonication, and repelleted; then the supernatant was discarded. This wash step was repeated three times before the liquid was discarded and the particles were resuspended in 0.1 M PBS at a concentration of 1 mg mL^−1^ using sonication and then stored at 4 °C.

### APTES amination

Freshly synthesised nanoparticles were suspended in 1 mL of ethanol plus 4% [v/v] (3-aminopropyl) triethoxysilane (APTES) and stirred at 200 rpm for 3 h at room temperature while stirring in a Falcon tube. The aminated particles were then transferred to a centrifuge tube (Corex) followed by 2× washes with ethanol and centrifuged at 11 000*g* for 25 min. The contents were then washed once using 2-(*N*-morpholino) ethanesulfonic acid (MES) buffer (pH 7.0) and then resuspended in MES buffer at the final concentration of 1 mg mL^−1^.

### Affimer production

Anti-CEA specific Affimer clones were identified using a ‘phage display library’ method as recently published by Shamsuddin *et al.*^30,31^ Out of the three CEA binding Affimers identified, clone II and III (molecular weight 12.5 and 12.6 kDa respectively) were chosen for this study having 9 and 10 distinct amino acid residues at the variable region, respectively. The anti-human cardiac myoglobin Affimer was used as a control. Anti-CEA (II and III) and control Affimer clone (molecular weight 12.5 kDa) DNA were isolated as previously described and the Affimer proteins were expressed from a pET11a vector in BL21 (DE3) *E.coli* cells. The *E.coli* cells were grown in Luria–Bertani broth medium containing 100 μg mL^−1^ carbenicillin until the growth was 0.8 at A_600_. Then cells were induced with 0.1 mM IPTG and incubated at 25 °C for 6 hours. The cells were harvested by centrifugation and lysed; the His_6_ tagged Affimers were purified by Ni^2+^–NTA affinity chromatography (Merck, New Jersey, USA). Pierce® immobilised tris (2-carboxyethyl) phosphine (TCEP) reducing gel was used to reduce Affimer disulphide bonds to free all thiol groups for subsequent maleimide coupling chemistry. TCEP gel (150 μL) was washed with PBS containing 1 mM edetate disodium (EDTA) three times followed by 4 μL of PBS containing 50 mM EDTA, followed by adding 150 μL of 0.5 mg mL^−1^ Affimer. The mixture was stirred at 20 rpm for 1 h, then centrifuged at 1000*g* for 1 min and finally the reduced Affimers were recovered from the supernatant.

### Silica nanoparticle Affimer conjugation

Fresh sulfosuccinimidyl 4-(maleimidomethyl) cyclohexane-1-carboxylate (SMCC) (6 mg) was mixed with 60 μg (1 mg mL^−1^) of polyclonal anti-CEA or anti-myoglobin Affimers and stirred gently at room temperature for 20 minutes. The reaction mixture was then added to 4 mL of 1 mg mL^−1^ aminated nanoparticles and stirred at room temperature for 2 h and then washed twice with PBS (6000*g* for 15 min) to remove unbound sulfo-SMCC. The nanoparticles were resuspended at 2 mg mL^−1^ and finally 0.1% (w/v) BSA was added. The nanoparticles were either stored in the dark at 4 °C or used immediately for *in vitro* experiments.

### Scanning electron microscopy (SEM)

SEM images were obtained with a field emission gun scanning electron microscope (FEG-SEM, LEO 1530 Gemini FEGSEM) fitted with an Oxford Instruments 80 mm X-Max SDD detector, Carl Zeiss.

### Spectrofluorometer measurements of silica nanoparticles

The fluorescence intensity of silica coated nanoparticles was quantified on a spectrofluorometer (Berthold Technologies Mithras LB 940 multimode microplate reader with Mikro Win 2000 software) with a halogen lamp intensity of 23 000 and excitation and emission spectra at 645 nm ± 30 nm.

### Affimer per nanoparticle quantification assay

Affimer tagged NPs were suspended in PBS at 1 mg mL^−1^, then mixed with 5 mL of 2-mercaptoethanol 1% (v/v) and incubated for 1 h at 37 °C. The suspension was then centrifuged at 12 500*g* for 30 min. The supernatant was recovered, then desalted using a Zeba spin desalting column (7K MWCO) to remove any remnants that might interfere with the fluorescent dye NanoOrange®. The released Affimers were quantified using a NanoOrange® protein quantitation kit.

Standard solutions of Affimer (0–2.5 μg) were prepared in 1× NanoOrange® reagent working solution from 10 μg mL^−1^ stock solutions. For sample analysis, 10 μl of each desalted solution was mixed with 240 μl of 1× NanoOrange® working solution. All standard and sample solutions were prepared in 500 μL tubes and incubated at 95 °C in a water bath for 10 min. All processes carried out were protected from light. The samples were allowed to cool down at RT for 20 min before 200 μL of each solution was transferred to a 96-well plate for fluorescence intensity measurement. The measurement was carried out with excitation and emission wavelengths of 485 nm and 590 nm, respectively. The fluorescence values of the standards and samples were subtracted from the value of the blank reagent. The corrected values were used in generating calibration curves using GraphPrism and linear fitting was performed.

### Dynamic light scattering (DLS)

The DLS measurement for nanoparticles was made using a Zetasizer Nano series Nano-ZS DLS system with a red (633 nm) laser (Malvern Instruments Ltd) at room temperature and in a small volume disposable cuvette. The polydispersity index (PDI) of the colloidal solutions was measured using DLS with a particle size analyser. The zeta potential or overall surface charge of each nanoparticle sample in solution (∼1 mg mL^−1^ in Millipore water) was determined using a Zeta Plus zeta potential analyser (Brookhaven Instruments Corp. Holtsville, NY).

### Nanoparticle-mediated fluorescence imaging *in vitro*

The HEK293 epithelial cell line, as a control, and the human colorectal cancer cell lines, LoVo, HCT116, and LS174T, were obtained from the American Type Culture Collection (ATCC). HEK293 cells were maintained in DMEM (1×) with GlutaMAX™-I (Gibco®) and 10% (v/v) FBS (Sigma life Science). Cancer cells were maintained in Advanced MEM (ATCC) for LoVo, F12K Nutrimix (Invitrogen, USA) for LS174T and RPMI 1640 (Invitrogen, USA) for HCT116. All cells were supplemented with 10% FCS and 1% l-glutamine at 37 °C in 5% CO_2_. Cells (9 × 10^4^) were seeded onto sterile glass coverslips (CellPath, Newtown Powys, UK) in a six-well plate (Corning) and incubated at 37 °C in 5% CO_2_ for 24 h. Culture media were discarded and cells were washed 2 times with PBS followed by addition of paraformaldehyde (4%, v/v) for fixation. Following incubation for 30 min at room temperature, the fixative was removed and cells were washed 3 times with PBS. BSA (0.1% (w/v), (EMD chemicals, San Diego, USA)) was added for 30 min then followed by washing 3 times with PBS. Anti-CEA or anti-myoglobin Affimer tagged nanoparticles (1 mg mL^−1^) were added to the wells and incubated for 24 h in the dark at room temperature. The nanoparticle suspension was discarded and the cells were washed 3 times with PBS and then coverslips were mounted onto glass slides using Depex (Waltham, Massachusetts, USA). The slides were left to cure overnight, then stored at 4 °C in the dark and imaged using confocal microscopy. Images were captured using a Nikon A1R-A1 confocal microscope (Nikon, Japan) with NHS Elements software (v 4.0). ImageJ v1.42q (NIH Freeware, USA) was used to quantify fluorescence.

### Nanoparticle internalisation and co-localisation *in vitro*

LoVo and LS174T cells (2 × 10^4^) were seeded onto sterile coverslips and allowed to adhere overnight. In the following day, cells were treated with fluorescein isothiocyanate (FITC) and CEA- or Myo-Affimer tagged-nanoparticles (1 mg mL^−1^) for 1, 4 and 24 hours. Cells were washed three times with PBS to remove nanoparticle suspension and incubated with LysoTracker™ Deep Red (Thermo Fisher) at 50 nM for 1 h. Following three washes with PBS, cells were fixed with 4% PFA. Following routine wash, the nucleus was counterstained with DAPI and mounted onto slides using mounting media (FluoroShield, Sigma). To monitor NP uptake, imaging was performed at 100× magnification using a fluorescence microscope.

### Photodynamic therapy and cell cytotoxicity *in vitro*

LoVo, LS174T, HCT116 and HEK293 cells were grown in two identical 6-well plates. Anti-CEA Affimer tagged nanoparticles and their respective controls at various concentrations (1–5 mg mL^−1^) were added to the wells and incubated for 24 h in the dark at room temperature. The nanoparticle suspension was removed after 24 h, and the cells were washed 3 times with PBS and then incubated with fresh media. The plates were then immediately placed on top of a light-radiating device (Avago Technologies, California, USA). Cells were treated with a light dose of 0.225–0.675 J cm^−2^, a peak wavelength of 600–700 nm and a spectral half-width of 12 nm, and then kept in the dark. Light dose was calculated based on treatments which lasted for 10–45 min at 0.25 mW cm^−2^. Control plates were kept in the dark with no light irradiation.

Stock solution of 3-[4,5-dimethylthiazole-2-yl]-2,5-diphenyltetrazolium bromide (MTT) tetrazolium salt MTT (Sigma) was prepared at 5 mg mL^−1^ in PBS and wrapped in foil to protect from light. The media, in which the seeded cells were grown, were replaced with 50 μL of 1 mg mL^−1^ working MTT solution and incubated in the dark for 3 hours. MTT solution was then removed and the dark blue formazan dye formed was dissolved in 100 μL of propan-1-ol. Optical density was measured using a microplate reader (Opsys MR™, Dynex Technologies Ltd, UK) at 570 nm.

### Cellular reactive oxygen species detection assay

Cells were seeded on a 96 well plate at 2.5 × 10^4^ cells per well and incubated for 24 h. Cells were then washed once using 1× buffer and then stained with 25 μM 2′,7′-dichlorofluorescin diacetate (DCFDA) in 1× buffer for 45 min at 37 °C. Cells were then washed once with PBS then incubated with functionalised silica nanoparticles for 24 h in the dark and then illuminated for 30 min. Immediately after illumination, the nanoparticle suspension was then discarded, 10 μM DCF-DA (Merck, New Jersey, USA) in Hank's balance salt solution (Merck, New Jersey, USA) was added for 30 min and incubated in a CO_2_ incubator, and then the mixture was washed with PBS. DCF fluorescence was observed using a confocal microscope.

### Fluorescence imaging *in vivo*

The *in vivo* experiments were conducted in a UK Home Office designated animal facility at Leeds Institute of Medical Research (University of Leeds, UK). The study was conducted in line with the UK Home Office regulations and in accordance with The Animals (Scientific Procedures) Act 1986, under a personal animal licence (Licence number: P93AOF172). BALB/c nu/nu female mice (4–6 weeks old) (Charles River, UK) were injected subcutaneously with 1.5 × 10^6^ LS174T cells to the right flank. Tumour xenografts were developed to nearly 10 mm in diameter within ∼10 days, and then mice were randomised to either CEA-targeted or control Affimer tagged-nanoparticles. Mice were injected with nanoparticles at 150 μL (suspended in sterile PBS at 2 mg mL^−1^ concentration) *via* the tail vein under general anaesthesia. Fluorescence images were captured using IVIS imaging (filters: excitation 672 nm, emission 694 nm; PerkinElmer, USA) under anaesthesia and then imaged at different time points. Living Image (v4.3.1, Caliper Life Sciences, USA) was used for fluorescence measurements (radiant efficiency in (p s^−1^ cm^−2^ sr^−1^)/(μW cm^−2^)) after calibration to background. *Ex vivo* fluorescence imaging was performed on all the resected tumour xenografts and the remaining organs.

### Photodynamic therapy *in vivo*

The PDT efficacy of Foslip encapsulated silica nanoparticles was evaluated in LS174T CRC xenograft models *in vivo*. Animal models were categorised into four treatment groups: (i) anti-CEA Affimer targeted NPs plus PDT laser treatment, (ii) anti-myoglobin targeted NPs plus PDT laser treatment, (iii) anti-CEA targeted NPs alone and (iv) anti-myoglobin targeted NPs alone. Tumour xenograft volumes, mouse weights and body conditioning scores were recorded before and after the PDT experiment. Mice were anaesthetised, then immobilised in plexiglass holders 24 h post intravenous injection and then irradiated at the xenografts using a 650 nm fibre-optic laser. The laser was positioned 18 mm directly above the skin, delivering a total light dose of 50 J cm^−2^, at a fluence rate of 60 mW cm^−2^ resulting in a total irradiation time of 14 min. Thermal imaging videos were recorded for 1 min at 0, 7 and 14 min during treatment for each mouse. Mice were maintained and monitored for 5 days post PDT treatment. Following completion of the experiment, mice were euthanised in accordance with Schedule 1 of the Animals (Scientific Procedures) Act 1986 and the tumour xenografts and organs were harvested. The efficacy of PDT was evaluated by histological analysis in harvested tissue.

### Statistical analysis

GraphPad Prism Version 9.0 (GraphPad Software, California, USA) was used for the statistical analysis of all the data. The difference between the groups was evaluated using Student's *t*-test and Wilcoxon signed-rank test.

## Author contributions

Y. S. K., P. A. M., T. A. H. and D. G. J. conceived and designed the experiments. E. A. and S. H. S. performed the Affimer expression and purification. M. I. K., T. M, N. L., A. P. and Y. S. K. performed the *in vivo* experiments and analysed the data. R. A.-M. performed fluorescence microscopy experiments. Y. S. K. performed all the other experiments and analysed the data. L. C. contributed to design of the *in vivo* experiments. A. P., D. T., L. C., D. G. J., T. A. H., P. A. M. and J. T. contributed to study design. All authors interpreted the results. Y. S. K., P. A. M., T. A. H. and D. G. J. co-wrote the manuscript. All authors discussed the results and commented on the manuscript.

## Conflicts of interest

The authors have no other relevant affiliations or financial involvement with any organisation or entity with financial interest in or financial conflict with the subject matter or materials discussed in the manuscript apart from those disclosed. No writing assistance was utilised in the production of this manuscript.

## Note after first publication

This article replaces the version published on 3 Nov 2023, which contained errors in Figure 5F.

## Supplementary Material

NR-016-D3NR04118B-s001
