# Supplementary material for: Photoactive imaging and therapy for colorectal cancer using a CEA-Affimer conjugated Foslip nanoparticle
Source: Nanoscale. 2023 Nov 3;16(14):7185–99. doi: 10.1039/d3nr04118b (PMC10993305; doi:10.1039/d3nr04118b)
Supplement: NR-016-D3NR04118B-s001 [file NR-016-D3NR04118B-s001.pdf]

Electronic Supplementary Material (ESI) for Nanoscale.
This journal is © The Royal Society of Chemistry 2024

This ESI replaces the original version published on 3 Nov 2023.

Title: Photoactive diagnosis and therapy for colorectal cancer using a CEA-Affimer conjugated Foslip nanoparticle

Supplementary Figures

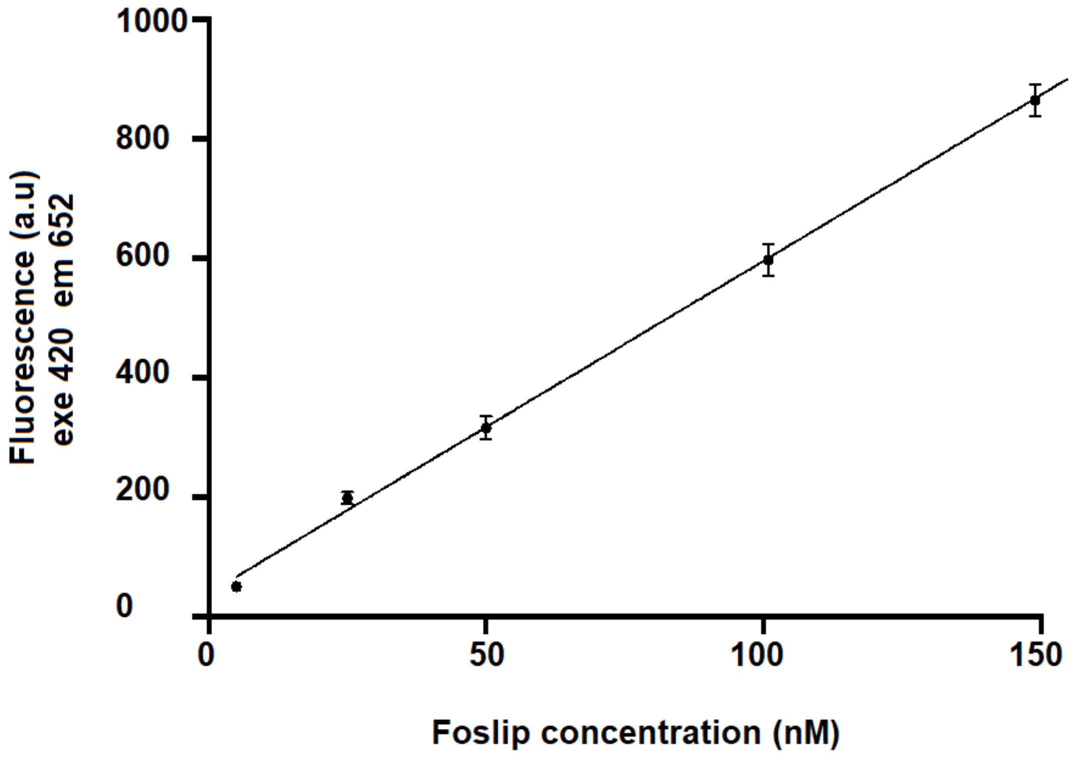

Sample	Fluorescence	Nanoparticle concentration (mg/ml)	Foslip Concentration used (nM)	EE (%)
1	950	1	200	82.1
2	970	1	200	80.9
3	989	1	200	83.2
		**Mean**	**200**	**82.2**

Figure S1. *Foslip encapsulation efficiency in silica nanoparticles synthesis.* A standard curve of absorbance was plotted for soluble Foslip. Foslip-loaded silica nanoparticles were manufactured using a water-in-oil microemulsion technique and suspended in water then readings were taken for samples from three separate batches of Foslip-loaded nanoparticles.

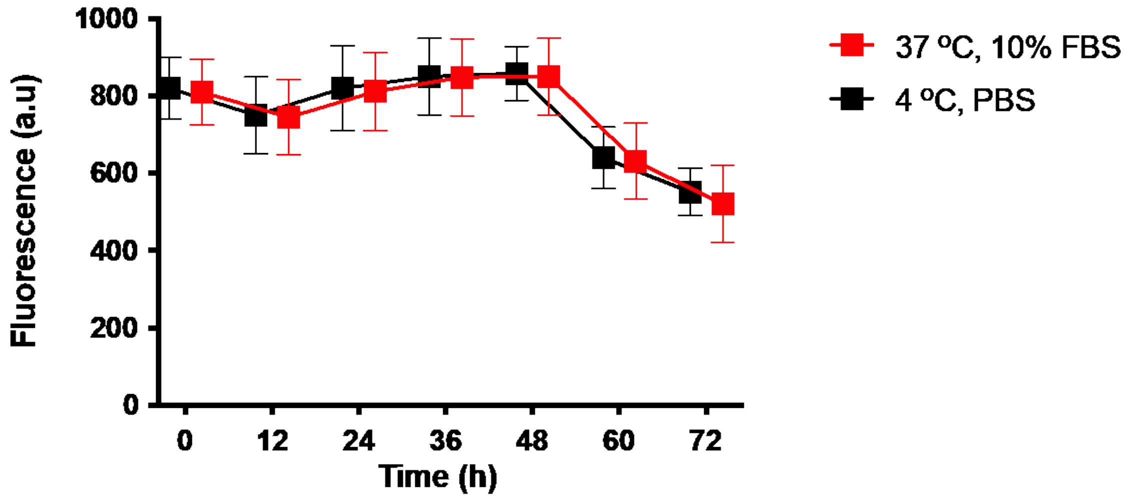

Figure S2. *Stability of Affimer tagged NPs at different time points.* Stability of Affimer tagged NPs monitored by fluorescence intensity against time from the point of freshly synthesised NPs (0 h). Data show mean from 3 biological experiments (SEM, n=3).

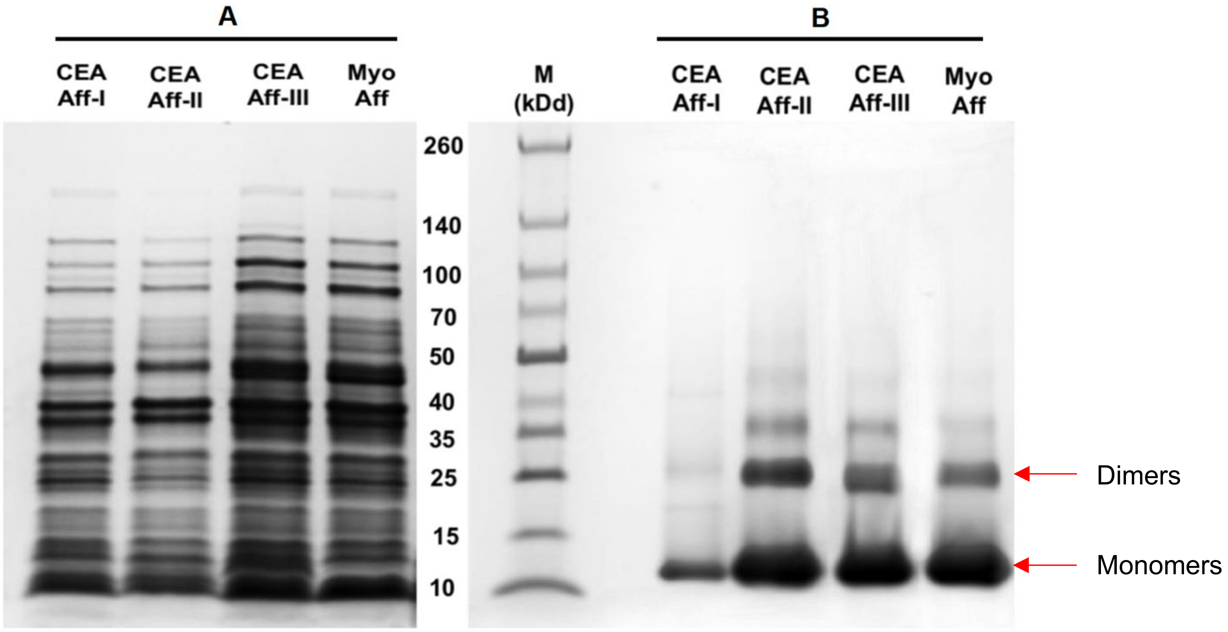

Figure S3. *SDS-PAGE gel of purified anti-CEA Affimers.* Gels show: (A); soluble fractions of each Affimer before Ni^{2+}-NTA resin purification; (B), purified Affimers after Ni^{2+}-NTA resin had been loaded. Five µl of each sample was added to each well and run in 4-15% (w/v) gradient gel under reducing condition. For all three anti-CEA Affimer (I-III), the elution bands migrated in the range between 10-15 KDa, suggesting successful Affimer monomers purification. The intensity of the bands observed indicated that anti-CEA Affimer I was less expressed than anti-CEA Affimer II and III despite using the same volume of eluant (5 µl). Affimer dimers were seen on the gel due to the presence of cysteine amino acid in the C-terminus. This is due to disulphide bond formation between two cysteine groups on two Affimers. The lanes denote: (M), protein marker in KDa; (CEA-Aff-I), anti-CEA Affimer I; (CEA-Aff-II), anti-CEA Affimer II; (CEA-III), anti-CEA Affimer III and (Myo-Aff), anti-myoglobin Affimer.

Table ST1: Outcome of anti-CEA and anti-myoglobin Affimers expression and purification.

Clone	Concentration (mg/mL)	Aggregation	Volume
Anti-CEA-I	0.5	Yes	1 ml
Anti-CEA-II	3.1	No	1 ml
Anti-CEA-III	3.7	No	1 ml
Anti-myoglobin	2.9	No	1 ml

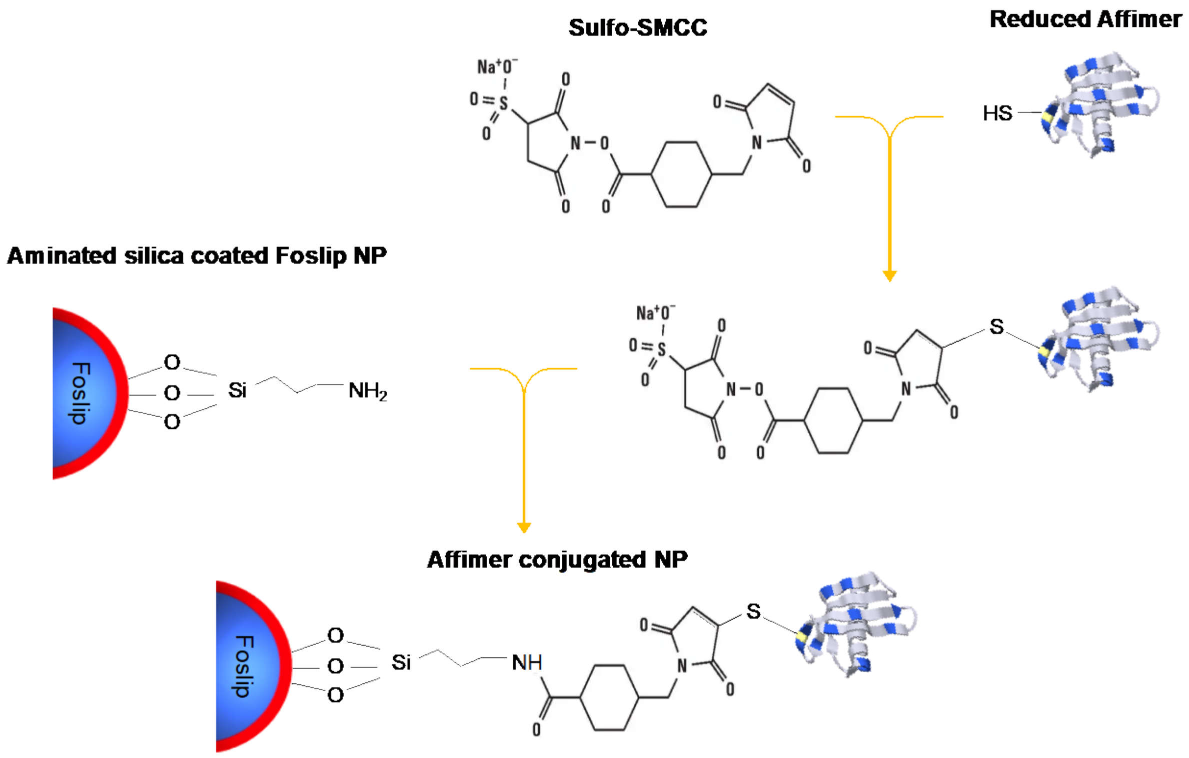

Figure S4. *Schematic of chemical linkage for Affimer tagged NP*. Sulfo-SMCC maleimide group reacts with the free sulfhydryl group on a reduced Affimer then conjugated to the aminated nanoparticle surface to form a stable amide bond. The blue colour on the reduced Affimer represents the 11 lysine residues at the surface of the Affimer scaffold whilst the yellow colour represents the cysteine at the C-terminal (pdp. 4N6T).

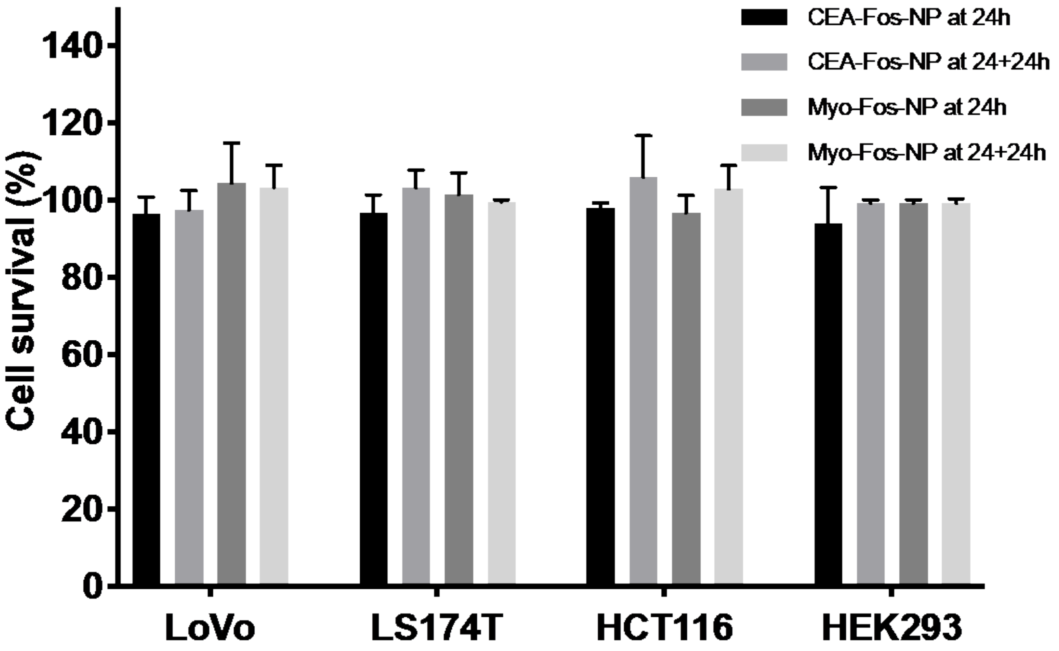

Figure S5. *Dark toxicity of Affimer-tagged nanoparticles in LoVo, LS174T, HCT116 and HEK293 cell lines.* Cells were incubated with 3 mg/mL concentrations for 24 h in the dark then washed and kept in free media for additional 24 h. Cells viability was quantified using MTT assay. Data denote mean cells viability from 3 biological experiments (SEM, n=3).

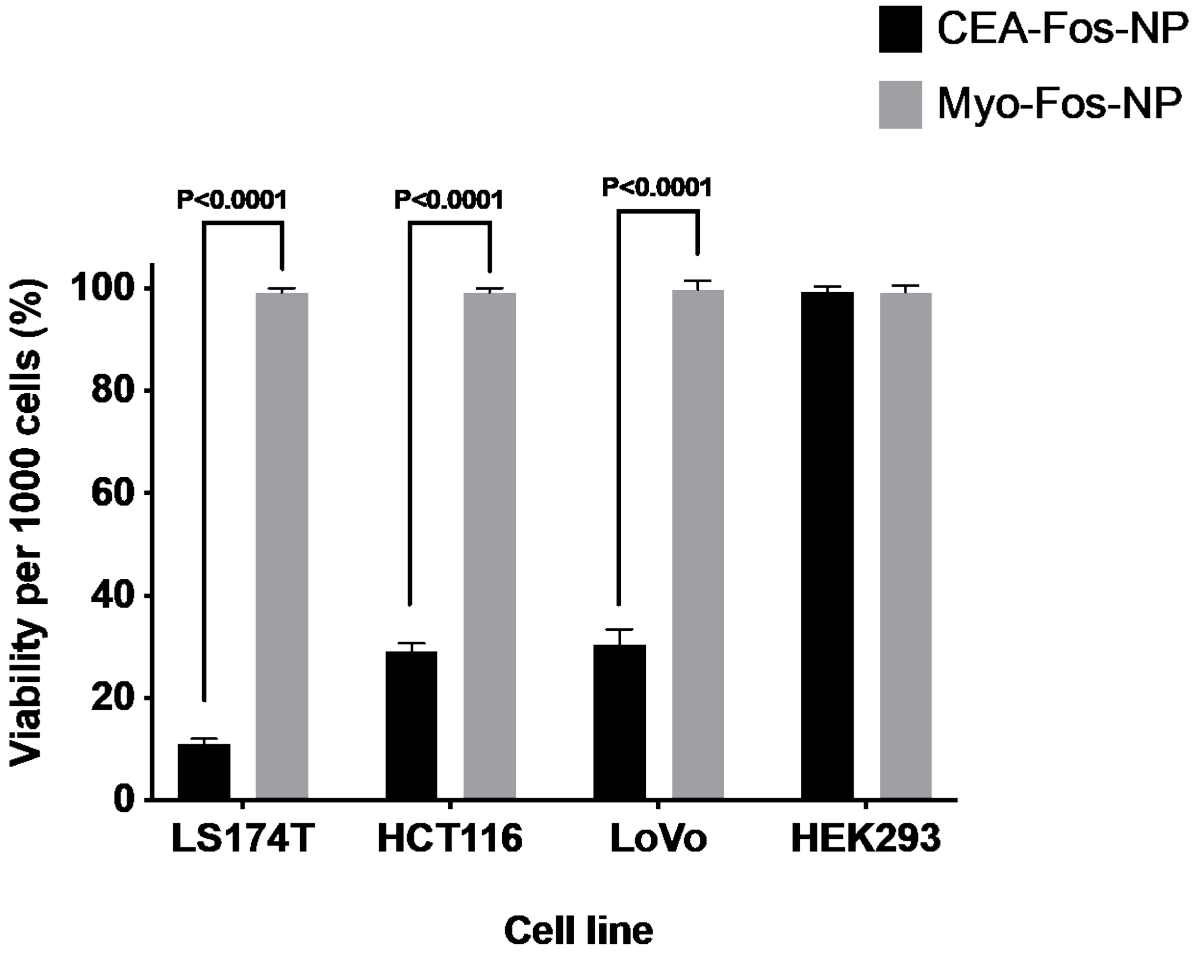

Figure S6. *Normalised PDT induced cytotoxicity per 1000 cells.* PDT effect on cell viability per 1000 cells in CEA-Fos-NP and Myo-Fos-NP at 2 mg/mL concentration. Cell viability was quantified using Trypan Blue assay. Data show mean cells viability from 3 biological experiments (SEM, n=3).

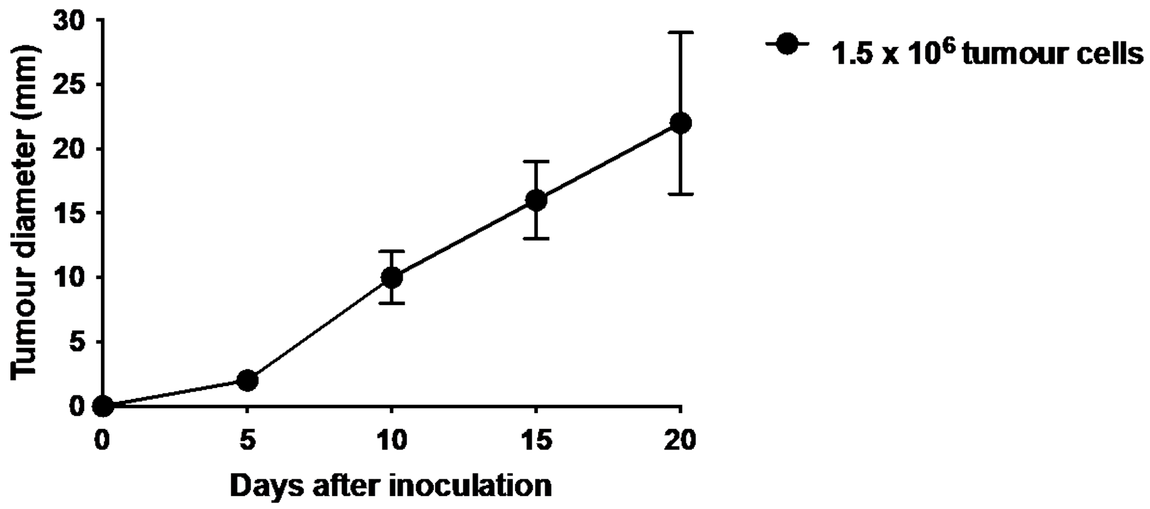

Figure S7. *LS174T tumour xenograft growth.* BALB/c nu/nu female mice (4-6 weeks old) were injected subcutaneously with 1.5 × 10^6 LS174T cells to the right flank. Tumour diameter was measured every 5 days. Data denote mean xenograft diameter from 3 mice (SEM, n=3).

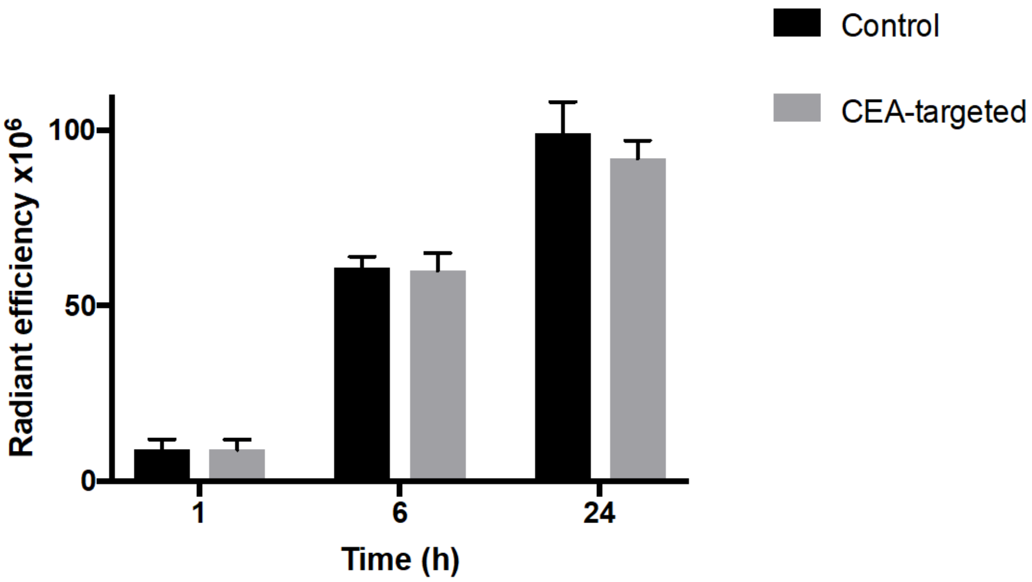

Figure S8. *Hepatic fluorescence following systemic delivery of control and CEA-targeted nanoparticles in vivo.* Data denote mean fluorescence (SEM, n=3). Control: anti-myoglobin Affimer tagged nanoparticles.

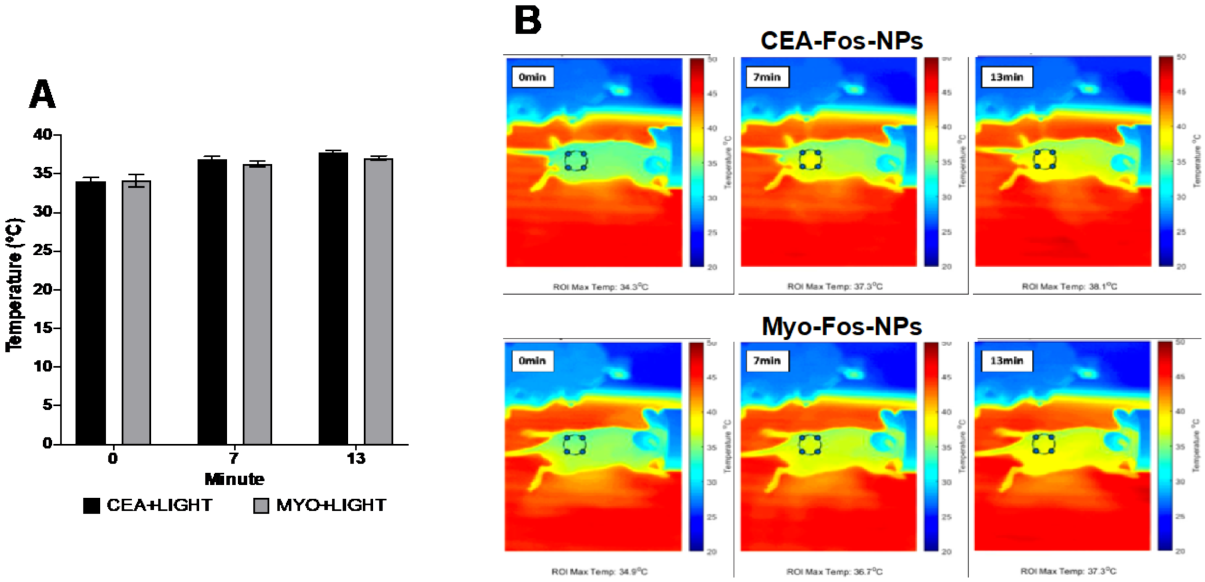

Figure S9. Thermal assessment during *in vivo* PDT. (A), Body temperature measurement at 0, 7 and 14 minutes. Data denote mean temperature (SEM, n=5). (B), Respective thermal images captured during laser irradiation. Thermal imaging videos were recorded for 1 min at 0 min, 7 mins and 14 mins during treatment for each mouse. ROI means region of interest. Scale bar represents temperature in ^{0}C degree.
